# Mechanisms of carbapenem resistance: exploring the complexities

**DOI:** 10.1093/jacamr/dlz013

**Published:** 2019-04-12

## Abstract

**Graphical Abstract:**
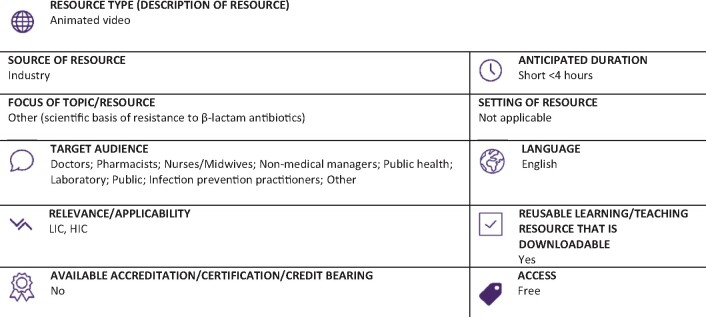

**Resource web link:** https://www.shionogi.com/wp-content/uploads/Mechanisms_of_Carbapenem_Resistance_Exploring_the_Complexities_1.mp4?_=2 (Full classification scheme available at: http://bsac.org.uk/wp-content/uploads/2019/03/Educational-resource-review-classification-scheme.pdf)

**WHO region and country (World Bank):** Western Pacific, Japan (HIC)

## Peer review commentary

This resource is an industry-prepared (Shionogi) animation that aims to explain the mechanisms of resistance to β-lactam antibiotics in Gram-negative bacteria, naming three priority groups (Enterobacteriaceae, *Pseudomonas* and *Acinetobacter*).

The 5 min video explains how β-lactam antibiotics work to kill Gram-negative bacteria. This is followed by an explanation of the three main mechanisms by which bacteria become resistant to β-lactam agents, namely β-lactamases, porin channels and efflux pumps. Details are provided about the classes of carbapenemases. References are provided at the end of the video.

The video works equally well on a smartphone as it does on a computer, although the quality of the picture does vary. There is minimal text on the screen and the video is narrated in English. The video is generalized to β-lactam agents rather than specific drugs, which makes it applicable to any resource setting.

You would need some scientific knowledge to fully understand the content of the video (i.e. the meaning of non-fermenters, Gram stain, components of a bacterial cell) but the resource may be useful to students, laboratory scientists etc.

This resource would be a useful teaching aid that could be incorporated into other educational resources.

